# Notes on American Opium from Vermont

**Published:** 1868-11

**Authors:** William Proctor


### Notes on American Opium from Vermont.

By William Proctor, Jr.

A few weeks ago my attention was called to a sample of "opium," by Mr. C. Wilson, of Monkton, Addison Co., Vermont, who said he had been requested by persons interested in the success of his enterprise to have it examined. On inquiry as to its origin, Mr. Wilson said it was of his own production in the neighborhood above mentioned, and that he had been engaged in the culture for several years, and that it was quite lucrative. After the weather was settled in the spring the seed of the opium poppy (Papaver somniferum,) was sown in ground prepared as for a garden, in which the plants grew vigorously, and about the middle of August the capsules attained their size. The collection of the juice was commenced at this time and continued until the first of September, when the whole plants were cut, bruised with a portion of alcohol to prevent fermentation, and then subjected to strong pressure; the juice thus obtained was evaporated to an extract, incorporated with the inspissated juice of the capsules, so that when finished the whole constituted a soft mass of pilular consistence and nearly homogenous texture, (except a few fragments of vegetable tissue,) possessing a strong narcotic odor almost precisely that of good ordinary opium, but not so decided, and a uniform dark brown color. Its reaction is acid. This year Mr. Wilson obtained 640 pounds of this opium from six and a quarter acres of land, being 100 pounds to the acre, for which he obtained prices varying from eight to ten dollars per pound from druggists and physicians in New England.

When macerated in water it soon breaks down and is readily extracted. The pulpy matter left from 100 grains after percolation with water until exhausted, amounted to 25 grains. One hundred grains carefully dried in a hot air bath weighed 84 grains, and hence contains 16 per cent. of moisture. Subjected to the action of diluted alcohol until exhausted, the residue weighed 13 grains. Treated with ordinary ether and dried, the moist opium lost 20 per cent. of its weight; but 16 per cent. of this loss is due to water in the normal opium, leaving the ethereal extract equivalent to 4 per cent. The ethereal solution had a light greenish color, due to chlorophylle. On evaporating the ether spontaneously, the residue consisted of numerous minute, well-defined crystals of narcotina, a greenish oleo-resinous matter, and the odorous matter of the opium. The crystals are nearly all prisms, with parallel sides and two-sided oblique terminations, and a few stellate groups occur. Separated and wiped, they afford an intense yellow color to nitric acid, and when treated with sulphuric acid followed by nitrate of potassa, they yield the usual deep red coloration of Orfila's test for narcotina. Benzine extracted 4.5 per cent. of green elastic caoutchouc matter containing narcotina. The aqueous and alcoholic solutions respond freely to the tests for *meconic acid.*

The morphia present was assayed by the process of Mohr.

100 grains of the moist opium (representing 84 grains dried) was exhausted with repeated portions of cold water and finally percolated, until four fluid ounces of infusion was obtained. This was boiled with 100 grains of lime previously slaked with some of the weaker liquid for fifteen minutes, filtered hot and the dregs percolated with boiling water till exhausted of the soluble matters of the opium. The alkaline infusion, slightly acidulated with muriatic acid, was evaporated to about half a fluidounce, and when cold neutralized with ammonia and filtered, to separate coloring matter, and then carefully evaporated to about 200 grains, and a slight excess of ammonia added whilst yet warm. After standing twelve hours the crystalline precipitate was carefully collected on a small tared filter, washed, dried, treated with ether and weighed 6·25 grs. This precipitate afforded the characteristic reactions of morphia with nitric acid and sesqui-chloride of iron.

Now from these results it must be inferred that this new kind of opium contains 6·25 per cent. of morphia in its moist commercial condition, or 7·44 per cent. when it is dry; and that it is much more soluble in water than ordinary opium, affording 75 per cent. of its weight to that fluid. The tincture made from it by the officinal process has the appearance and odor of ordinary laudanum, but of its therapeutic character in relation to Smyrna opium I am wholly uninformed. Now there need be no hesitation in saying that this opium is below the standard of the Pharmacopœia. The maker appears to be entirely candid and honest in his conduct of the process, and the fault is in his not knowing the real character of the substance he is dealing with, and the importance in medical and hygienic points of view that it be parallel in strength with fair Turkish opium, to obtain and deserve the confidence of physicians, apothecaries and druggists. It is probable that the pure exudation from the capsules unmixed with any foreign matter rarely reaches us in the opium market, and there may be less impropriety in employing the inspissated juice of the poppy than the various matters that are introduced at Smyrna and elsewhere, to give consistence to the too soft exudation from the capsule and increase the volume of the product. The fact that 640 pounds of an opium, containing between six and seven per cent. of morphia, was produced in a few weeks after the poppy attained its proper size, from six and a quarter acres of land, in a climate as far north as Vermont, by a moderate force, seems to warrant the belief that, under intelligent regulations, the culture of opium might be effected in this country so as to be a profitable crop. The need of assaying it would be imperative until its physical characters became sufficiently well established to be depended on by commercial dealers.

We would advise Mr. Wilson, he knowing the amount of extract he adds, to reduce its quantity so that the pure juice of the capsules may bear a larger proportion to the gross amount produced. Probably one-half less would make the result nearer commercial opium, containing 10 per cent. of morphia.

There are various experiments going on at the south and west, in Mississippi and elsewhere, this season, but as yet the results have not reached me. The subject is sufficiently important to claim the attention of the American Pharmaceutical Association, and if experimenters throughout the country will communicate their results to the writer with a clear statement of the processes of culture and preparation employed, he will engage to give a faithful report of them to the next meeting at Chicago. It would be best to accompany each communication, if any are sent, with about half an ounce of the product, fairly representing the gross amount produced by the sender.—*Journal of Pharmacy.*

The *Medical Gazette* is cautioning ladies against the dangers of using cosmetics. The large quantity of lead employed in the composition, with other poisonous ingredients should be a warning to them.

